# Editorial: Structural understanding of the functional consequences of missense mutation

**DOI:** 10.3389/fgene.2023.1325326

**Published:** 2023-11-07

**Authors:** Jian Tian, Christopher D. McFarland, Jaie Woodard

**Affiliations:** ^1^ Institute of Animal Science, Chinese Academy of Agricultural Sciences, Beijing, China; ^2^ Department of Genetics and Genome Sciences, Case Western Reserve University, Cleveland, OH, United States; ^3^ Department of Biomedical Engineering, University of Michigan, Ann Arbor, MI, United States

**Keywords:** missense mutation, protein structure, functional consequence, mutation analysis, protein function

## Introduction

Missense mutations are genetic changes that modify the amino acid sequence of proteins. Recent advances in predicting the structure of proteins have made significant strides, yet much is still unknown about the effects of missense mutations on protein structure and function, particularly in terms of their role in evolution and disease. A single amino acid substitution can drastically alter a protein’s stability, location, expression, catalytic activity, and capacity to interact with other proteins or ligands, which can have a significant impact on cellular physiology (Cheng et al., 2023).

This Research Topic focuses on gaining a better understanding of missense mutations through protein structural, sequence, and phylogenetic analysis. This Research Topic contains five papers that further the Structural Understanding of the Functional Implications of Missense Mutation. These studies introduce a technique to detect the effect of mutations on protein folding, analyze the effect of mutations of genes (TP53 and NRAS) on carcinogenesis, analyze mutations of the virus SARS-CoV-2, and introduce a method for the evaluation of the Catalogue Of Somatic Mutations In Cancer (COSMIC) signatures. Contributions to this Research Topic cover one or more of the research areas indicated by the letters.

## Approach to detect mutations that alter protein folding stability

Mutations within a protein’s coding sequence can often cause it to misfold, which can have detrimental effects on cells. However, it is difficult to measure the destabilizing effects of different mutations because current methods rely on essential proteins as sensors, and misfolded proteins can disrupt their function. To address this, the study by Quan et al. presents a novel *in vivo* selection screen called Intra-FCY1, which can be used to identify mutations that cause misfolding of proteins in yeast. Intra-FCY1 utilizes two complementary fragments of the toxic yeast cytosine deaminase Fcy1, into which a model protein (yellow fluorescent protein, YFP) is inserted. When YFP folds correctly, the Fcy1 fragments associate and become toxic in media containing 5-fluorocytosine, which inhibits growth. However, mutations that cause YFP to misfold prevent Fcy1 toxicity, allowing mutant strains to rise to high frequency in growth competition experiments. Misfolding character was confirmed through Western blotting, which found that all selected variants reduced the solubility of YFP. This system can be used to study the relative stability of mutant versions of any cellular protein, independent of localization. The approach described can identify novel mutations that cause misfolding, demonstrating the potential for this method to explore the relationship between protein sequence and stability.

## Analysis of the effect of somatic mutations (TP53 and NRAS) on carcinogenesis

This section contains two papers that investigate the effect of alterations in NARS and TP53 on carcinogenesis. The two studies introduce methods to identify detrimental SNPs or characteristics that are associated with the relevant mutations.

The first paper focuses on the analysis of the deleterious single nucleotide polymorphisms (SNPs) of the NRAS gene (Behairy et al.). The NRAS gene is a well-known oncogene that plays a major role in carcinogenesis. Mutations in the NRAS gene have been linked to various types of human tumors, so it is essential to identify the most damaging single nucleotide polymorphisms (SNPs) in the NRAS gene in order to understand the key factors relevant to tumor pathogenesis. This study used sequence and structural approaches to filter out the most deleterious SNPs and structural biology methods and docking tools to analyze structural details. After analyzing the missense SNPs with six *in silico* tools, 17 mutations were identified as the most damaging. All SNPs, except S145L, were found to reduce NRAS stability, and all SNPs were located on highly conserved residues and important functional domains, except R164C. Furthermore, all mutations, except G60E and S145L, showed a higher binding affinity to GTP—the ligand needed for mitotic signaling, suggesting an increased malignancy tendency. The remaining 14 mutations were predicted to increase the risk of carcinogenesis, with 5 mutations (G13R, G13C, G13V, P34R, and V152F) expected to have the highest risk. Further experimental studies with all these 14 mutations could provide new insights into the pathogenesis and management of different types of tumors.

The second study presents a series of bioinformatic analyses to explore the characteristics of TP53 mutations in hepatocellular carcinoma (HCC) (Yang et al.). Gene mutations, copy number variations, tumor mutational burden and microsatellite instability, protein-protein interaction network, differential gene expression, and functional enrichment of TP53 mutations were analyzed. Mutations in the missense category accounted for a large proportion of HCC mutations, with single nucleotide polymorphisms being highly common and C > T being the most typical single nucleotide variation. In the TP53 mutant group, the tumor mutational burden, drug sensitivity, ESTIMATE score, and stromal score decreased significantly (*p* < 0.001, *p* < 0.05, *p* = 0.038, and *p* < 0.001 respectively). Cystoscope software identified ten hub genes, such as CT45A1, XAGE1B, CT55, GAGE2A, PASD1, MAGEA4, CTAG2, MAGEA10, MAGEC1, and SAGE1. This research sheds light on TP53-mutated HCC and offers new perspectives for personalized treatments for HCC, which is beneficial for prognosis forecasting.

## Mutation analysis of the virus SARS-CoV-2

This paper studies 4,622 SARS-CoV-2 sequences from Bangladesh in order to gain a better understanding of the virus and develop strategies to control the pandemic (Shishir et al.). The results show that the sequences belonged to 35 major PANGO lineages (a SARS-CoV-2 genetic taxonomy), with Delta accounting for 39%, and 78% from four primary lineages. Furthermore, Dhaka was identified as the center of viral transmission, and the virus was seen to be moving back and forth across the country. Additionally, 7,659 unique mutations were identified, with an average of 24.61 missense mutations per sequence. The analysis of genetic diversity and mutation patterns revealed that eight genes were under negative selection pressure to eliminate deleterious mutations, while three genes were under positive selection pressure. These findings, in combination with an ongoing genomic surveillance program, can help us to better comprehend the evolution and pandemic characteristics of SARS-CoV-2 in Bangladesh.

## Method for analysis of mutational signatures extraction via archetypal analysis

This study presents a systematic analysis of the *de novo* extraction process through simulations using the most recent version of the COSMIC signatures (Pancotti et al.). Utilizing a novel approach to archetypal analysis, it was discovered that 29 archetypes could recreate the profile of all COSMIC signatures with a cosine similarity of at least 0.8. These archetypes tend to group similar original signatures that have the same origin or related biological processes. This research is beneficial in encouraging the development of new *de novo* extraction techniques that can eliminate redundancy of information while still maintaining proper biological interpretation.

All these papers adopt a multi-scale approach to examining the mutations based on their structure, sequence, and phylogenetic characteristics. Nevertheless, it is still a great challenge to comprehend or predict functional, health, and fitness consequences given only knowledge of the identity of a missense mutation. Therefore, extensive and diverse data regarding each mutation and its phenotype must be compiled, and Artificial Intelligence techniques may be leveraged to bridge this gap in the future.
